# Supplementary material for: Are carbon emissions trading and green financial instruments synergistic? -Comprehensive quantitative research based on content analysis
Source: PLoS One. 2024 Mar 7;19(3):e0298601. doi: 10.1371/journal.pone.0298601 (PMC10919601; doi:10.1371/journal.pone.0298601)
Supplement: S1 Data — (ZIP) [file pone.0298601.s001.zip › Data/地级市Z_F工作报告.zip!/░▓╗╒/╨√│╟╩╨/└┤╫╘╣1⁄2╓┌║┼í╛┬φ┐╦╩2╛▌═°í┐_2005.docx]

各位代表：

现在，我代表市人民政府向大会作政府工作报告，请予审议，并请市政协委员和其他列席人员提出意见。

一、开拓奋进，快速发展的2004年

过去的2004年，在省委省政府和市委的领导下，全市人民坚持以邓小平理论和“三个代表”重要思想为指导，认真贯彻落实党的十六大和十六届三中、四中全会精神，主动顺应宏观调控的新变化，积极应对经济紧运行的新挑战，不断深化融入苏浙沪经济圈的发展战略，经济社会发展取得了一系列新的成就。全年生产总值达到235.7亿元，比上年增长10.4%，人均生产总值突破1000美元。完成财政总收入16.85亿元，增长32.2%，其中地方财政收入8.97亿元，增长22.4%。城镇居民人均可支配收入7153元，增长10.8%。农民人均纯收入2844元，增长13.7%。全面建设小康社会迈出坚实的一步。

一年来，我们主要做了以下六个方面的工作：

第一，注重调整和优化经济结构，提高经济增长质量和效益。农业基础地位得到加强，农业生产稳步回升。全年农业增加值比上年增长6.3%，粮食总产达到115.6万吨，增长20.9%。农业产业化经营扎实推进，农业标准化生产取得新进展。全市国家和省级农业产业化龙头企业发展到14家，有20个农产品获得省级名牌农产品称号，103个农产品获得无公害、绿色和有机食品标志认证，总数居全省第一位。农村劳动力转移步伐继续加快，非农产业收入进一步增加。工业经济在调整中保持较快增长，质量和效益继续提高。规模以上工业增加值增长30.1%，工业固定资产投资增长72.4%；宣城海螺水泥、宣城电厂、司尔特40万吨复合肥等20个重点工业项目进展顺利，新和成皖南药业、哈威车轮、三狮水泥等项目建成投产。服务业发展加快，启动了整合“红色”资源、创建“红色”品牌工作，旅游、通信、商贸、房地产等消费热点继续升温。全年旅游总收入8.7亿元，增长29%；社会消费品零售总额72.3亿元，增长9.2%。金融保险业稳健运行，年末金融机构各项存贷款余额分别比年初增加24.8亿元和10.1亿元。民营经济发展势头较好，全市个体工商户发展到8.6万户，私营企业达到5112家，民营经济占全市经济总量的份额达到54.5%。

第二，加快推进改革开放，不断增强经济发展活力。企业改革继续向纵深推进，列入市考核的334户国有集体企业，有300户完成改制；列入省考核的60户国有集体企业，全面完成改制任务。农村税费改革和粮食补贴方式改革不断深化，取消农业税附加，降低农业税税率，减轻了农民负担。公共财政支出改革取得新的进展，市直单位财务管理进一步规范，乡财县管改革全面推开。市县两级政府机构改革顺利实施，事业单位改革逐步深入，粮食流通体制、金融保险和投融资体制等各项改革稳步推进。积极开展面向苏浙沪等沿海发达地区为重点的区域经济合作，强力推进招商引资。全年实际利用外资5866万美元，比上年增长41.6%；实际到位省外资金44.08亿元，总量居全省第三位。对外贸易稳步增长，全市外贸进出口总值达到1.56亿美元，增长25.7%，其中出口1.31亿美元，增长50.1%。开发区和工业园区建设取得新的进展，落户企业不断增多，投资规模进一步扩大。

第三，千方百计扩大投资，努力改善经济发展环境。结合全面建设小康社会规划目标和第一阶段建设任务，启动实施了六大产业基地和八大基础工程。全年完成固定资产投资110.5亿元，比上年增长47.8%。水利基础设施建设步伐加快，水阳江、青弋江流域防洪规划已经水利部批准，宣城市城市防洪、水阳江综合治理等重大水利项目前期工作取得新的进展。交通“十三路八桥”工程有七路七桥基本完成，318国道广祠段高速公路建成通车，溧黄高速公路前期工作全面展开，县乡公路“通达工程”加速推进。宣杭铁路复线、宣城50万伏变电所等一批重点工程项目正在抓紧建设。宣城市城市总体规划修编工作已经完成，城市近期建设规划启动实施，皖东南救灾物资储备中心一期工程、市消防指挥中心和大坝塘游园等项目建成使用，敬亭山东大门、市区“两纵两横”道路改造等工程基本完成，市疾病控制中心、昭亭路改造、梅溪路立交桥、府山广场、城市天然气利用等工程进展顺利。市区交通秩序和市容市貌综合整治工作扎实推进。

第四，高度关注民生，着力解决关系群众利益的突出问题。全面落实就业和再就业扶持政策，率先在全省建立了统一的城乡就业服务体系，市场化就业机制初步形成。全年新增就业岗位2.13万个，安置下岗失业人员1.2万人，城镇登记失业率控制在4%以内。企业职工基本养老保险市级调剂金制度全面实施，养老、失业、医疗等社会保险覆盖面继续扩大。农村特困群众生活救助制度初步建立，城乡低保工作得到加强。工伤保险启动实施，新型农村合作医疗试点进展顺利。扶贫开发工作扎实推进，贫困地区群众生产生活条件进一步改善。社区建设得到加强，区街经济有了新的发展。认真解决土地征用、城镇拆迁、企业改制过程中侵害群众利益的突出问题，基本完成建筑领域农民工工资清欠任务。积极履行社会管理职能，突发公共事件应急预案正在抓紧制定。加强非典防治工作，采取果断措施,及时控制禽流感疫情。大力实施食品药品放心工程，扎实开展生产生活资料市场、农用车非法载客和市区禁放烟花爆竹等专项整治活动。深入开展以交通、煤矿为重点的安全整顿，有效防止了重特大事故的发生。加强社会治安防控体系建设，广泛开展遵纪守法好公民、见义勇为先进个人评选和公安机关大练兵活动，严厉打击各种刑事犯罪和经济犯罪，“平安宣城”建设全面展开。

第五，坚持经济社会协调发展，全面推进各项社会事业。认真组织实施国家和省重大科技计划项目，全市有4个项目获省科学技术奖，经省认定的高新技术企业达到28家，高新技术产品46项，新增民营科技企业14家。切实把教育放在优先发展的战略地位，进一步整合教育资源，着力抓好中小学布局调整和危房改造工作，基础教育得到巩固，高中阶段教育继续扩大，职业教育、民办教育和高等教育有了新的发展。人口与计划生育综合改革顺利通过国家中期评估，人口出生率、出生人口性别比等主要指标继续保持全省先进水平。公共卫生体系建设得到加强，城乡卫生条件不断改善。文化事业发展加快，申报获得“中国文房四宝之乡”称号，成功举办了首届中国·宣城文房四宝之乡文化旅游节、中国曹禺戏剧奖第十一届“飞彩杯”全国百优小品大赛和第二届宣城艺术节。广播电视覆盖面继续扩大，自办节目水平不断提高，数字电视正式开通。全民健身运动广泛开展，竞技体育在全国和省级比赛中取得较好成绩。严格国土资源管理，经营性土地“招拍挂”和土地整理、开发复垦工作取得积极成效。“生态宣城”建设步伐加快，实施了一批生态示范项目，小化工、小钢铁等专项整治取得阶段性成果。精神文明创建活动逐步深入，群众思想道德素质和文明程度有了新的提高。民族、宗教、外事、侨务、对台工作不断进步，审计、物价、气象、地震、文物、地方志和新闻出版等各项工作迈出新步伐。经济普查摸底工作全面完成，市档案馆晋升为省一级国家综合档案馆。妇女儿童、老年人、残疾人权益得到保障。国防后备力量建设和“双拥”工作进一步加强。

第六，大力推进依法行政，注重提高施政水平。积极贯彻落实行政许可法和国务院依法行政实施纲要，全面清理行政许可项目、规定和实施主体，清理结果全部向社会公布。坚持政风评议，推行政务公开，逐步完善市县两级政务服务中心的服务功能，评选表彰了一批人民满意的公务员和公务员集体。“四五”法制宣传教育有效推进，扎实开展创建“民主法治示范村”、“法律进社区”和“送法下乡”活动。坚持走访人大代表和政协委员联系制度，自觉接受人大法律监督和政协民主监督，市政府全年共办理人大议案2件、代表建议50件，政协委员提案102件。及时听取各民主党派、工商联和无党派人士的意见与建议，加强与工会、共青团、妇联等群众团体的联系，重视发挥舆论和社会监督作用。进一步加强和改进信访工作，积极开展争创“六有六无”乡镇活动，群体性事件和集体上访得到及时稳妥处理。注重从源头上预防和治理腐败，广泛开展反腐倡廉警示教育活动，配合上级有关部门依法查处了“4·28”等违法违纪案件，解决了一些群众反映强烈的热点问题。

各位代表，过去的一年，我们坚持把融入苏浙沪经济圈作为经济发展的主战略，不断加大招商引资力度，新的经济增长点逐步增多；全面落实中央、省支持农业发展的各项政策措施，有力促进了粮食增产和农民增收；主动顺应土地、财税、信贷和行业政策调整，坚决清理和压缩不符合国家产业政策的投资项目，加强经济运行调节，有效缓解了经济发展中的突出矛盾，实现了速度与结构、质量、效益的统一。这些成绩是省委省政府和市委总揽全局、正确领导的结果，是全市人民凝心聚力、开拓奋进的结果。在此，我谨代表市人民政府，向全市广大工人、农民、知识分子、干部、驻宣解放军指战员、武警官兵、政法干警，各民主党派、各人民团体和其他各界人士，致以崇高的敬意！向关心、支持宣城现代化建设的中央和省属各单位，向在我市创业的境内外投资者、建设者，表示衷心的感谢！

经济社会发展的好形势，增强了我们继续前进的信心和勇气。但我们也清醒地认识到，在加快发展的道路上还有许多困难和问题。一是经济总量小、工业化水平低、结构层次低的局面还未改变，制约经济发展的体制性矛盾依然突出，经济快速增长的基础还不够稳固。二是宏观经济环境不确定因素增加，土地、资金等要素供给偏紧。三是农民增收依然困难，就业和再就业矛盾比较突出，影响社会稳定的因素较多。四是财政收支平衡压力仍然较大，城市建设和管理的任务较为艰巨。五是政府职能转变还不够到位，工作作风需要继续改进，廉政建设有待进一步加强等。对于这些问题，我们将予以高度重视，并采取切实有效措施，认真加以解决。

二、统筹发展，扎实做好2005年的各项工作

今年是全面实现“十五”计划目标、衔接“十一五”发展的重要一年，也是本届政府任期届满之年。扎实做好以经济建设为中心的各项工作，对于巩固和发展宏观调控成果、保持经济社会良好发展势头、圆满完成本届政府的任期目标，具有十分重要的意义。当前，世界经济继续保持增长，国内经济仍处于上升周期，国家促进中部地区崛起的政策和长三角经济加速融合，给我们带来了难得的发展机遇。更为重要的是，经过改革开放二十多年来的建设，特别是随着“融入”战略的逐步深化，我市人均GDP已达到1000美元这个标志性界线，迈入工业化加速推进的新阶段。在这样一个重要的发展阶段，我市具备的各种优势正在加速转化，多年积累的能量正在加速释放，显现出较大的发展潜力和增长空间。我们将进一步坚定加快发展的必胜信念，进一步形成克难攻坚的强大合力，切实把加快发展的强烈愿望转化为开拓奋进的具体行动，推动经济社会更快更好地向前发展。

根据中央和省委省政府的部署，结合我市实际，今年政府工作的总体要求是：以邓小平理论和“三个代表”重要思想为指导，认真贯彻落实党的十六大和十六届三中、四中全会精神，牢固树立科学发展观和正确的政绩观，始终把加快发展作为第一要务，继续坚持融入苏浙沪经济圈不动摇，坚持工业化战略不动摇，坚持招商引资不动摇，坚持发展民营经济不动摇，大力实施“一主两翼”发展战略，切实把经济发展的着力点放到扩大总量、调整结构、深化改革、转变经济增长方式上来，进一步提高人民生活水平，促进经济快速协调健康发展和社会全面进步。

按照上述总体要求，今年全市经济和社会发展的主要预期目标是：生产总值增长10%左右，财政收入增幅略高于生产总值增幅；固定资产投资增长25%左右；城镇居民人均可支配收入增长7%左右，农民人均纯收入增长6%左右；城镇登记失业率控制在4.5%以内；人口自然增长率控制在5‰以内。为实现上述目标，市政府将团结和依靠全市人民，着力抓好以下几个方面的工作：

（一）实施“一主两翼”发展战略，争创产业发展新优势

大力实施以工业为主体、以效益农业和旅游业为两翼的“一主两翼”发展战略，扎实推进宣城经济融入苏浙沪经济圈的历史进程，实现经济跨越式发展。加速工业化是我市全面建设小康社会的首要任务，必须进一步强化工业意识，坚定不移地走新型工业化道路，以信息化带动工业化，加快总量扩张和产业升级。立足现有工业基础，做大做强支柱产业和骨干企业，大力扶持汽车零部件、机械电子制造、医药化工、建材原材料、农产品加工等支柱产业发展，开工建设宣城电厂、宣城海螺水泥等一批重点工业项目，确保司尔特40万吨复合肥等项目建成投产，加快推进飞彩、古泉等大中型企业的战略性重组。着力抓好中鼎汽车零部件基地、新鸿发汽车注塑件、裕华纺织等重点技改项目和精方药业现代中药制造等高新技术产业化项目的实施。继续加快开发区和工业园区建设，着力培育一批高成长性的企业群体，逐步使开发区和工业园区成为吸纳境内外投资、承接苏浙沪等沿海发达地区产业转移的主要基地。全面实施“中小企业促进工程”，引导中小企业向专精特新方向发展，大力发展配套经济，加快构筑“小产品、大规模，小企业、大协作”的块状经济格局。注重节约能源和资源，禁止新上污染项目，坚决淘汰浪费资源、污染环境的落后工艺、设备和产品。继续强化企业管理，加强煤电油运和资金供应的协调，切实改善工业经济运行环境。

按照统筹城乡发展的新思维谋划农业，坚持用工业的理念经营农业，促进农业增效、农民增收。稳定粮食播种面积，加强优质粮油基地建设，推进粮食产业化进程。加大农业结构调整力度，突出抓好畜牧养殖、特经作物、经济林果、特种水产等主导产业的发展，加快南漪湖综合开发，积极扩大家禽、生猪、茶叶、烟叶、蚕桑、苎麻、竹类、山核桃、宣木瓜、河蟹等优势农产品的种养规模，打造基地化的特色农业。继续抓好国家级家禽、茶叶标准化综合示范区和省级农业标准化示范县建设，健全农产品质量安全检验、检测和动物防疫体系，引导农民按标准化组织生产，提高农产品质量和市场竞争力。实施“强龙工程”，主攻农产品综合开发和精深加工，扎实推进农业产业化经营，促进农产品增值。整合支农资金，重点扶持和威、太阳等一批农业产业化龙头企业，增强辐射带动能力。进一步完善农业服务体系，继续加强农村经济信息网建设，大力发展农产品行业协会、农村专业合作组织和农民经纪人队伍，注重培育营销大户和农村致富带头人，提高农民的组织化程度。

坚持用大旅游观念和系统工程的方法发展旅游业，加速建设苏浙沪地区旅游观光的“后花园”。整合旅游资源，创新旅游品牌，重点开发建设以泾县新四军军部旧址和“皖南事变”发生地为龙头的红色旅游，以敬亭山、胡氏宗祠、胡适故居、江村、桃花潭、查济古民居为代表的文化旅游，以扬子鳄湖、太极洞、障山大峡谷、青龙湖、南漪湖为主体的生态景观旅游，增强旅游产品的吸引力。积极争创全国“优秀旅游城市”，精心打造“中国文房四宝之乡”城市名片，在宣城市区着手建设文房四宝一条街，并逐步配套建设体现“文房四宝之乡”文化内涵的广场、雕塑、博物馆、图书馆、美术馆等，扩大宣城的知名度。加强以道路交通为重点的旅游基础设施建设，提高旅游从业人员的整体素质，改善旅游接待环境和服务水平。加快旅游商品开发，着力发展体现地方特色的旅游工艺品、纪念品和食用品。强化旅游宣传促销，加强与“两山一湖”和“长三角”旅游圈的沟通合作，实现“点线旅游”与“板块旅游”互动，形成规模效益。在大力发展旅游业的同时，坚持提升和发展并举，继续加快商贸流通、交通运输等传统服务业的改组改造，积极培育汽车住房、教育培训、医疗保健等消费热点，推广连锁经营等新型业态，大力发展信息、咨询、中介、社区服务等现代服务业，不断提高服务业占全市经济的比重。

（二）继续抓好招商引资工作，提高对外开放水平

招商引资是我市扩大投资需求的重要渠道，也是推动经济增长和社会进步的支撑力量。今年将在已有基础上，继续升温加压，强力推进，确保引进外资6000万美元，引进省外资金45亿元。充分发挥企业特别是优势骨干企业的主体作用，鼓励大中型企业利用优质资产、优势产品、优良品牌等对外招商，力争在大企业、大项目合资合作上取得突破。继续面向苏浙沪，主动对接“珠三角”，突出工业招商，加大基础设施、农业、服务业和社会事业招商引资力度，提升招商引资规模和水平。不断改进招商引资方式，把工作侧重点由“招商引资”向“招商选资”转变，积极运用委托招商、以商招商等灵活有效的方式，推动招商引资工作专业化、制度化和规范化。完善招商引资责任制，健全工作协调机制，强化跟踪服务，提高合同履约率、资金到位率和项目成功率。更加重视安商扶商工作，大力优化招商环境，提高服务水平，切实增强招商引资的吸引力。

融入苏浙沪经济圈，既是宣城经济发展的一项长期战略，也是当前十分紧迫的任务。要以更加积极的姿态推进“融入”，加快在规划、产业、市场、基础设施等方面的对接，重点加强制造业、旅游业、农业等领域的合作与交流。认真落实各项出口促进政策，大力扶持和培育具有出口竞争能力的重点产品、骨干企业和优势企业，积极鼓励和支持地产品出口，努力扩大农业创汇、旅游创汇和劳务创汇，力争全年外贸进出口总值达到1.9亿美元，其中出口1.6亿美元。

（三）积极推进体制和机制创新，大力发展民营经济

继续深化企业产权制度改革，坚持分类指导，规范操作，力争年内全面完成国有集体企业改制任务。进一步完善法人治理结构，推进公司制企业规范化运作。充分利用资本市场，加快培育上市梯队，扩大企业直接融资规模。加强国有资产监督管理，确保国有资产保值增值。深化农村税费改革和粮食流通体制改革，全面取消农业税，继续实行粮食直补、良种补贴，进一步规范“一事一议”行为，切实减轻农民负担。稳定和完善农村土地承包关系，加快农村土地承包经营权流转。改革农村小型水利产权制度和管理体制，推进社会化办水利。采取有效措施，逐步化解村级债务。完善配套措施，积极稳妥地实施事业单位改革，进一步深化科技、教育、文化、卫生等社会事业改革。

认真抓好财政金融和投融资体制改革。依法加强税收征管，强化政府非税收入管理，保持财政收入稳定增长。完善公共财政体系和“乡财县管”体制，继续强化综合预算、部门预算和国库集中支付等制度，严格控制一般性财政支出，确保工资按时足额发放，确保社会保障支出，确保机关工作正常运转。加强对重点项目资金和各类专项资金的审计监督，保障财政资金有序运行。积极支持金融保险业改革，完成农村信用社以县为单位的统一法人工作。继续推广农户小额信贷和联户担保贷款，稳步推进农业保险，切实搞好农村金融服务。深化投融资体制改革，积极引导社会投资，推进重大基础设施和公用事业项目投融资方式创新。

毫不动摇地鼓励和支持民营经济发展。全面放开民间资本准入领域，进一步完善和落实相关政策，引导民间资本进入支柱产业、高新技术产业、现代农业和服务业。鼓励民营企业参与国有集体企业改革和基础设施、公用事业建设，积极扶持科技型、外向型、吸纳下岗人员就业型和农产品加工型民营企业的发展。加强和改进对民营企业的协调服务，落实公平待遇，建立完善融资担保、人才开发、技术创新、创业辅导等社会服务体系，切实保护私有财产权和民营企业的合法权益。

（四）加快基础设施和城镇建设，提升县域经济发展水平

扎实推进“861”行动计划，继续加快在建项目和已落实项目建设。重点抓好招商引资和利用国债资金安排的交通、水利、电力、通信等基础设施建设，确保工程进度和质量。加快推进“十三路八桥”工程，抓紧实施215省道广宁线、322省道宣水线和323省道鸿旌线建设，全面完成214省道望十线二期工程和322省道泾茂段建设任务，力争开工建设溧黄、宣铜、宣千高速公路。加强农村公路建设和管理，积极推进“乡乡通等级油路”建设和“村村通油路”试点。继续做好协调和服务工作，确保宣杭铁路复线年内建成通车和宣城50万伏变电所竣工送电。大力推进宣城市区和宁国、郎溪、绩溪、泾县等城市防洪工程建设，实施并完成以广德卢村、宣州塘埂头等水库为重点的除险加固工程，认真做好防汛抗旱工作。扎实开展水阳江综合治理、芜太运河宣城段等重大项目的前期工作，加强农村“六小工程”建设。继续加快通信光缆传输网、数据接入网和宽带城域网建设。

认真组织实施宣城市新一轮城市总体规划和近期建设规划，抓紧编制各类专项规划、分区规划和详细规划。基本完成梅溪路立交桥、昭亭南路、昭亭北路建设和天然气门站及主要城管网建设，整治叠嶂路、环城西路、宣南路和梅溪路，改造宛溪路，重建凤凰桥，积极筹建绩溪路西段和东门公跨铁立交桥，配套建设汽车客运站、停车场、污水处理厂、垃圾处理厂和医疗废弃物处理中心。积极稳妥地推进旧城改造，着手整治1—2条老城区干道，并由市区两级共同整治主要背街后巷道路，逐步扩大城市园林、绿地、广场面积，改善市民居住条件。全面推进社区建设，深化城市社区管理体制改革，增强社区服务功能。以创建省级文明城市为目标，深入开展城市环境综合整治，完善长效管理机制，提高城市品位。继续加快各县市城区和中心镇建设，强化产业支撑，繁荣城镇经济。

进一步加强对县域经济发展的指导和支持，完善县域经济考核评价体系，促进基础较好的地方率先发展，水平中等的地方迅速崛起，发展相对滞后的地方尽快赶上，努力形成县域经济竞相发展、全面发展的良好格局。继续以发展民营经济和中小企业为主，积极发展生态经济、旅游经济，突出发展农产品加工业、劳动密集型产业和配套产业，培育和增强县域经济的内生力量。市本级和宣州区要集中力量加快工业区、商贸区、旅游区的开发建设，大力提升加工制造业、现代服务业和农业的发展水平，市区联动，加快构筑全市经济中心的地位。

（五）始终坚持以人为本，促进社会和谐稳定

继续做好就业再就业工作。坚持把加快发展作为解决就业再就业问题的根本途径，大力发展非公有制经济和以社区服务业为重点的第三产业，努力增加就业岗位。实施积极的就业政策，认真落实税费减免、小额贷款、岗位补贴等促进就业的各项政策，完善就业培训和服务体系，鼓励自谋职业和自主创业，促进企业下岗失业人员实现再就业。加大再就业援助力度，大力开发公益性岗位，切实帮助困难群体特别是“4050”人员实现再就业。继续抓好劳务输出组织网络、劳动力市场和劳务培训基地建设，扩大劳务输出，统筹城乡就业。

进一步完善社会保障体系。依法推进社会保险扩面和社保关系接续工作，提高非公有制企业职工参保率。强化社保基金征缴，继续抓好基本养老保险市级调剂金制度的实施，确保养老金、失业金按时足额发放。加快社区劳动保障平台建设，推进企业退休人员移交社区管理和服务工作。认真实施工伤保险，完善失业保险和医疗保险制度，稳步扩大新型农村合作医疗试点范围。继续做好城乡低保和特困救助工作，支持有条件的地区适当调高低保标准，努力实现低保资金社会化发放。落实社会救助制度，做好救灾救济工作。加强劳动合同管理、劳动保障监察和法律援助工作，保障职工和弱势群体的合法权益。加快经济适用房和廉租房建设，逐步缓解中低收入家庭住房困难。

切实解决事关群众利益的突出问题。以“整村推进”工作为重点，扎实抓好扶贫开发和综合治理，努力改善贫困地区群众的生产生活条件。探索建立失地农民生活保障制度，认真处理好土地征用、城镇拆迁、企业改制、非法排污等涉及群众利益的问题，坚决制止拖欠和克扣农民工工资、教育乱收费等行为。加强价格监管，深入开展生产生活资料市场整治，大力实施食品药品放心工程，加快建立市场监管的长效机制，进一步规范市场经济秩序，建设“信用宣城”。继续强化安全生产监督和管理，扎实开展煤矿、非煤矿山、道路交通等重点行业安全整治，有效防止重特大事故发生，确保人民生命财产安全。

加快“平安宣城”建设。坚持“打防结合、预防为主”的方针，扎实推进社会治安防控体系建设，依法严厉打击各种犯罪活动。积极做好“四五”普法检查验收工作，广泛开展多种形式的法制宣传教育活动，加强基层司法所和人民调解组织建设。加大《信访条例》宣传力度，引导群众以理性合法的形式表达利益要求，认真落实信访工作的各项制度，依法及时合理地处理群众反映的问题，积极预防和妥善处置群体性事件，切实维护社会稳定。

（六）贯彻落实科学发展观，统筹发展各项社会事业

大力发展科技教育事业。依法推进科技进步与创新，完善科技进步奖励制度，积极扶持和引导科技企业发展。加强产学研结合，促进科技成果转化和高新技术产业化，提高科技进步对经济增长的贡献率。完善以县为主的农村义务教育管理体制，鼓励和发展社会力量办学。继续实施中小学危房改造和布局调整，努力改善办学条件。加强教师队伍建设和培训，进一步提高基础教育和高中阶段教育的质量。大力发展职业教育、成人教育和高等教育，加快培养技能型人才。开工建设青少年活动中心，积极筹建职业技术学院新校区。全面落实人才工作的各项政策，推动人才工作体制创新，努力形成育才、聚才、用才的新机制。

创新文化事业管理体制。加强乡镇文化站和市皖南花鼓戏剧团基地建设，广泛开展群众性文化活动，促进文化事业和文化产业发展。加强公共卫生设施建设，完善疾病信息网络和预防救治体系，切实抓好血吸虫病、结核病、艾滋病防治工作，全面完成市疾病控制中心建设。积极筹建市体育中心，广泛开展全民健身运动，提高竞技体育水平。加快发展广播电视事业,开工建设市广电中心，继续推进农村广播电视村村通工程。深入开展精神文明创建活动，加强社会公德、职业道德和家庭美德教育，提高全民素质。推进村民自治、民主管理，认真组织第六届村民委员会换届选举。依法保护妇女儿童、老年人和残疾人合法权益，支持工会、共青团、妇联等群众组织依照法律和章程独立自主地开展工作。认真落实党的民族、宗教、外事、侨务和对台政策，继续做好新闻出版、地震、气象、档案、文物、地方志等工作，全面完成经济普查任务。加强国防教育，支持国防建设，提高民兵预备役、人防和“双拥”工作水平。

加强人口、资源和环境保护工作。继续深入推进人口与计划生育综合改革，加强计生队伍建设和基层基础工作，稳定低生育水平。认真做好新一轮土地利用总体规划的修编工作，进一步落实耕地保护制度，切实保护基本农田。严格实行定额供地管理，制定和落实土地投资强度标准，提高土地利用率。依法保护和合理开发自然资源，全面完成生态市建设规划和宣城市城市环境规划编制，继续推进森林生态网络和生态示范区、生态县建设，加快建设“生态宣城”。以小化工、小钢铁、小造纸和燃煤锅炉为重点，强化污染整治，积极推广清洁生产，大力发展循环经济。认真做好“十一五”规划和2020年远景目标编制工作，努力提高规划的预见性、科学性和指导性。

三、加强政府行政能力建设，努力提高施政水平

做好今年各项工作，对政府自身建设提出了新的更高要求。我们将认真贯彻落实党的十六届四中全会精神，以提高政府行政能力为核心，大力推进科学行政、民主行政、依法行政，努力提高政府施政水平。

加快转变政府职能。按照建设公共服务型政府的要求，继续推进政企分开、政事分开，切实把政府职能转到经济调节、市场监管、社会管理和公共服务上来，着力解决政府职能的“缺位”、“越位”和“错位”问题。大力推进社会管理体制创新，建立健全国民经济和社会安全预警机制，加快自然灾害、事故灾难、公共卫生和社会安全等危机防控体系建设，完善矛盾调处、社会治安综合治理和城乡社区管理机制，切实增强保障公共安全和处置突发事件的能力。继续深化行政审批制度改革，加强市县两级政务服务中心建设，全面推行首问责任、办事限时和并联审批等制度，着力营造良好的经济发展环境，提高群众对政府工作的满意度，努力打造信用政府。

全面推进依法行政。深入贯彻落实行政许可法和国务院依法行政实施纲要，切实把政府管理工作纳入规范化、法制化轨道。坚持把科学的发展观和正确的政绩观贯穿于政府工作的全过程，加快完善公众参与、专家论证和政府决策相结合的决策机制，推进决策的科学化、民主化。自觉接受市人大、市政协和广大群众的监督，认真办理人代会议案、人大代表建议、意见和政协委员提案，主动听取各民主党派、工商联、无党派人士及各人民团体的意见和建议。理顺行政执法体制，开展综合执法试点，着力解决有法不依、执法不公、多头执法等群众反映强烈的问题。加强行政执法监督、行政投诉办理和行政复议工作，严格落实行政执法责任制、过错追究制，切实做到有权必有责、用权受监督、侵权要赔偿。深入开展廉政建设和反腐败斗争，建立健全教育、制度、监督并重的惩治和预防腐败工作体系，坚决查处各种腐败行为，努力打造廉洁政府。

切实提高行政效能。主动适应加强和改善宏观调控的要求，加强工作谋划，创新工作思路，切实增强政府工作的前瞻性、主动性和针对性。在政府机关深入开展保持共产党员先进性教育活动，充分发挥党组织的核心领导作用、战斗堡垒作用和党员的先锋模范作用，引导广大公务员树立正确的理想信念和高尚的道德情操，保持人民公仆本色，提高为民服务本领。扎实开展机关“效能建设”活动，有效防止行政不作为和乱作为。切实加大工作管理和落实的力度，着力解决管理松懈、纪律松弛和作风不实、办事不公等问题。坚持深入基层、深入群众，认真调查研究，掌握真实情况，把群众关注的热点、经济社会生活的难点和实际工作的薄弱点，作为工作落实的重点。深入推进政务公开，加快电子政务建设，增强政府工作的透明度。加大政务督办力度，强化目标考核管理，加强行政效能监察，确保政令畅通。继续开展政风评议活动，坚决纠正部门和行业不正之风，努力打造高效政府。

各位代表，新的一年，充满新的机遇，面临新的挑战，孕育新的希望。让我们紧密团结在以胡锦涛同志为总书记的党中央周围，始终坚持以邓小平理论和“三个代表”重要思想为指导，全面落实党的十六大和十六届三中、四中全会精神，在省委省政府和市委的坚强领导下，励精图治，团结拼搏，为加快发展、富民强市、全面建设小康社会而努力奋斗！
